# Transcriptomic and metabolomic analyses reveal the flavor of bitterness in the tip shoots of *Bambusa oldhamii* Munro

**DOI:** 10.1038/s41598-023-40918-8

**Published:** 2023-09-08

**Authors:** Yulian Jiao

**Affiliations:** Wenzhou Key Laboratory of Resource Plant Innovation and Utilization, Zhejiang Institute of Subtropical Crops, Zhejiang Academy of Agricultural Sciences, Wenzhou, 325005 Zhejiang China

**Keywords:** Computational biology and bioinformatics, Plant sciences

## Abstract

The young bamboo shoot of *Bambusa oldhamii* (green bamboo) has a good taste and is rich in nutrition and widely used in traditional Chinese cuisines. But the shoots flavor of *Bambusa oldhamii* changed from deliciously sweet to a little bitter when the shoots grew from underground to aboveground. In this paper, we explored the bitterness chemicals of the green bamboo shoot when growing from underground to aboveground using transcriptome and metabolome techniques. Finally, several bitter chemicals were mined out counting for the flavor transformation, such as Solanidine, Amygdalin, Salicin, Arbutin, and others. The transcription factor family of AP2/ERF plays the main role in key bitter chemical regulation via correlation analysis. Moreover, the pathway of Biosynthesis of phenylpropanoids might be the key pathway in the formation of the bitter chemicals in green bamboo shoot development.

## Introduction

The basic tastes, bitterness, sourness, sweetness, umami, and saltiness could be perceived and discriminated by humans. Plants protect themselves against being eaten by secreting natural pesticides and other toxins. Humans reject foods that are perceived to be excessively bitter^1^. Among bitter-tasting compounds are amino acids and peptides, sulfimides, ureas and thioureas, esters and lactones, terpenoids, and phenols and polyphenols. High-molecular-weight (> 500) polyphenols are also known as plant tannins^1^. Whereas lower-molecular-weight phenolic compounds tend to be bitter, higher-molecular-weight polymers are more likely to be astringent^1^. Polyphenols vary from simple phenolic molecules such as phenolic acids to highly polymerized compounds with molecular weights of greater than 30,000 Da such as tannins, and about 8000 phenolic structures existed in the plant kingdom. The polyphenolic compounds include simple phenols, benzoquinones, phenolic acids, acetophenones, phenylacetic acids, hydroxycinnamic acids, phenylpropenes, coumarins/isocoumarins, chromones, naftoquinones, xanthones, stilbenes, anthraquinones, flavonoids, lignans/neolignans, lignins^2^. The food flavonoids include chalcones, dihydrochalcones, aurones, flavones, flavonols, dihydroflavonol, flavanones, flavanol, flavandiol or leucoanthocyanidin, anthocyanidin, isoflavonoids, bioflavonoids, proanthocyanidins or condensed tannins^2^. Flavonoids (e.g., catechin, epicatechin, gallocatechin) are the monomeric constituents of condensed tannins^2^.

The following are some bitter phytonutrients in common plant foods: flavanones (naringin), flavones (tangeretin, nobiletin, sinensetin), flavonols (quercetin), flavans (catechin, epicatechin, epicatechin gallate, epigallocatechin, epigallocatechin gallate), phenolic flavonoids (catechin mono- and polymers; catechin polymers (tannins, astringent); polyphenols, isoflavones (genistein and daidzein, genistin, and daidzin), limonoid aglycones (limonin, nomilin, and limonin glucoside), glucosinolates (sinigrin, progoitrin, glucobrassicin), isothiocyanates (allyl-isothiocyanate, 3-methyl-sulfinylpropyl isothiocyanate, benzyl isothiocyanate, benzyl isothiocyanate, 4-methylsulfinyl butyl isothiocyanate, phenylethyl isothiocyanate)^1^. The accumulation of flavanones and flavones was influenced by AP2/ERF family via regulating type IV chalcone isomerase in citrus^3^. Cucurbitacins are triterpenoids that confer a bitter taste in cucurbits such as cucumber, melon, watermelon, squash, and pumpkin. Naringin was synthesized in the phenylpropanoid pathway and could treat human metabolic disorders^4^. The flavor of bitterness in fruits and vegetables was regulated by transcription factors, MYB and bHLH transcription factors might affect the fruit tasty of citrus by influencing the gene expression in the flavonoid pathway. In melon, CmBr (Bitter root) and CmBt (Bitter fruit) encode bHLH transcription factors that regulate Cucurbitacin B (Cu B) biosynthetic genes^5^. MYB transcription factor in hops could influence the production of flavonols but not prenylflavonoids or bitter acids^6^. Bitter acids (e.g. humulone) are important factors in the bitter flavor of beer, formed from acyl-CoA precursors, which might be regulated by MYB, WRKY, and other transcription factors^7^. In grapevine, the bHLH transcription factor plays roles in Proanthocyanidins (PAs), which determine the astringency and bitterness of red wines^8^.

Green bamboo (*Bambusa oldhamii* Munro), whose shoots emerged between 30/25 and 25/20 °C (Day/Night), indicating warm temperature positive effect on green shoot emergence^9^. And running bamboos grow faster than clumping bamboo^9^ when came out of the ground. Usually, juvenile shoots utilize as popular food items, a major component of their traditional cuisines^10^. The presence of high content of protein, amino acids, minerals, fiber, carbohydrates, and low fat makes the bamboo shoot one of the widely acclaimed nutrient-rich food items^10^. Moreover, raw, boiled, and fermented shoots of different bamboo species vary in nutrient composition^10^. Bamboo shoots are low in fats and cholesterol contents, but very high in potassium, carbohydrates, and dietary fibers^11^. The freshly harvested shoot is cream-yellow, has a strong fresh shoot smell, and is sweet. The chemicals of taxiphyllin, homogentisic acid, total sugar content, and total amino content like aspartic acid (Asp), glutamic acid (Glu), glycine (Gly) and tannin contents contributed to the flavor and taste of green bamboo shoots^11^. The metabolites concentration of L-phenylalanine, L-ornithine, adenine, and guanosine were increased as the bitterness level increased^12^.

The clumping bamboo *Bambusa oldhamii* has a geographical preference that limited its growth area in the southeast region of China. In suitable locations, every summer the delicious edible rhizome buds (developed into bamboo shoots) would be enlarged and stretched out from underground to aboveground. The best delicious tasty, crispy, and nutritious bamboo shoots are usually at the stages of just coming out of the surface, at this stage, the rhizome buds are usually enlarged enough, and the taste is good, the shoots at this stage are usually picked in the early morning, and transport to markets on sale in the morning. However, when the bamboo shoots stretched out from the soil surface, the sheaths color of the bamboo shoots would be changed to green with days, and bitterness tasty would be formed in the tip of the bamboo shoots. How is the special bamboo shoot tasty phenomenon formed by *B. oldhamii* from underground to aboveground? The special biological question and phenomenon interested us in deep searching using transcriptome and metabolome techniques. Combination analysis of transcriptomic and metabolic to reveal special biological questions had been widely used. In walnuts, the genes involved in raffinose and hydrolysable tannins biosynthesis had been reported, the enzyme of SDH and UGT metabolite 3-dehyydroshikimic acid to ellagic acid and play an important role in tannins biosynthesis^13^. In this research, several bitter chemicals (such as Solanidine, Amygdalin, Salicin, Arbutin, and others) were mined out to account for the flavor transition of bamboo shoots from underground to aboveground with transcriptome and metabolome profiling. Correlation analysis showed that the AP2-ERF-ERF transcription family might play a major role in the formation of bitter chemicals.

## Results

### Phenotypes and physicochemical properties of green bamboo shoots

**Figure 1:**
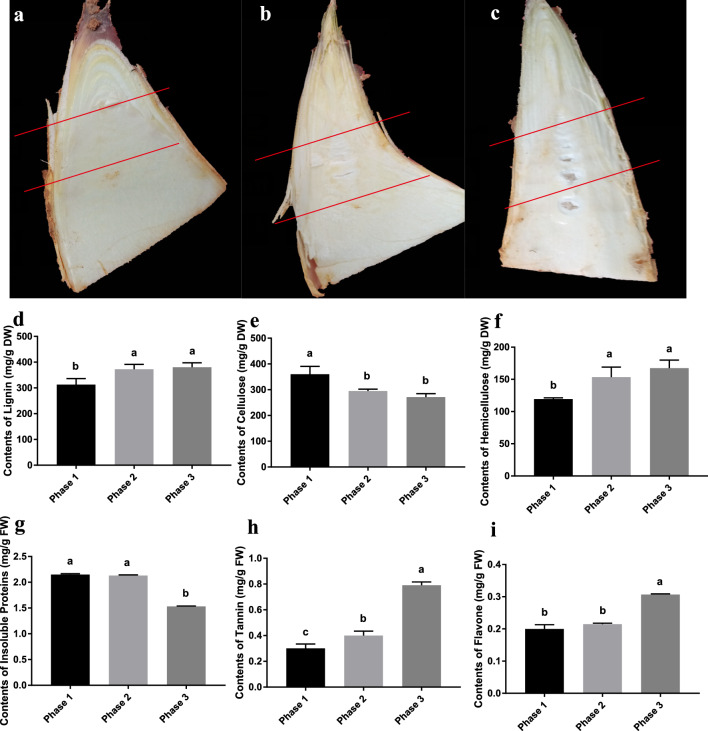
Phenotypes and physicochemical properties of three green bamboo shoots phases. (**a**) phase 1, the longitudinal section of bamboo shoots was underground; (**b**) phase 2, the longitudinal section of bamboo shoots have just stretched out of the ground; (**c**) phase 3, the longitudinal section of bamboo shoots whose top have stretched out of the ground and explored in sunlight for days; (**d**–**i**) the contents of Lignin, Cellulose, Hemicellulose, Insoluble Proteins, Tannin, Flavone in three green bamboo shoots developing phases respectively.

Usually, the green bamboo shoot has its best taste when growing underground which has a deliciously sweet, crisp, and tender texture. However, when the green bamboo shoot tip erupted from the ground and growing in aboveground for several days, the flavor of the green bamboo shoot would change to a little bitterness and a rough texture. The tip portion of three developing phases of green bamboo was used for searching the flavor changes in our research (Fig. 1a–c).

The texture from smooth and tender to rough and a little tough are usually caused by diet fibers and proteins. The contents of lignin were significantly increased in the second and third phases (Fig. 1d), and the cellulose contents were higher in the first phase (Fig. 1e), however, the contents of hemicellulose were lower in the first phase (Fig. 1f), The contents of insoluble proteins were significantly decreased in the third phase (Fig. 1g). The flavor from tiny sweetness to little bitterness is usually caused by bitter chemicals, like tannins and flavones. The contents of tannins were significantly increased with the three developing phases of green bamboo shoots (Fig. 1h). Flavone contents were significantly high in the third phase than in the first and second phases (Fig. 1i). In this article, we focus on the bitter taste changes, especially mining out the bitter chemicals for interpretations.

### Transcriptome basic profiles

A total of nine RNA-seq libraries were constructed and sequenced, All the reads from each library were quality controlled to ensure had a good quality of Q20 and Q30, and the GC contents in each library were above 51% (Table S1). All the clean reads were assembled with Trinity (v2.6.6), finally acquired 218,301 transcripts with 1500 bp of N50 and 596 bp of N90, and 67,399 Unigenes with 1445 bp of N50 and 460 bp of N90.

**Figure 2:**
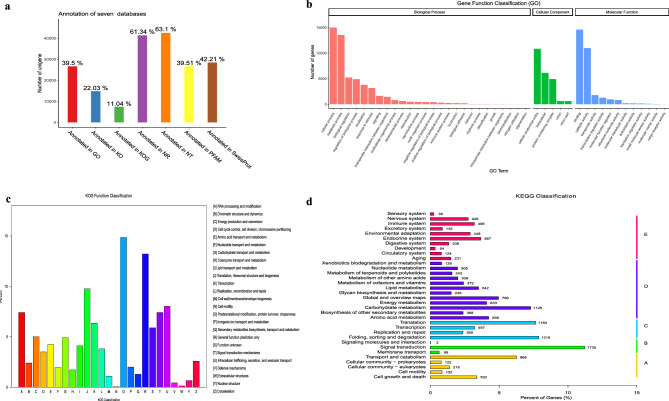
The statistic annotation and function classification of all the Unigenes. (**a**) The statistic annotation of seven databases; (**b**) The GO function classification of all Unigenes; (**c**) The KOG function classification of all Unigenes; (**d**) The KEGG classification of all Unigenes.

All the Unigenes were annotated in seven databases, about 39.5%, 22.03%, 11.04%, 61.34%, 63.1%, 39.51%, and 42.21% were annotated in GO, KO, KOG, NR, NT, PFAM, and SwissProt, respectively (Fig. 2a). The results of GO functional classification showed that in Biological Process, the term of cellular process and metabolic process occupied the most; in Cellular Component, the term of cellular anatomical entity, intracellular, and protein-containing complex occupied the majority; in Molecular Function, the term of binding and catalytic activity occupied the most (Fig. 2b). In the KOG Function Classification, the genes were more on the function of [O] posttranslational modification, protein turnover, chaperones; and [J] translation, ribosomal structure and biogenesis (Fig. 2c). In the KEGG^14,15^ classification, the genes were more on the function of signal transduction (1735), folding, sorting and degradation (1219), translation (1184), carbohydrate metabolism (1128), and transport and catabolism (968) (Fig. 2d).

### Transcriptome DEGs analysis

**Figure 3:**
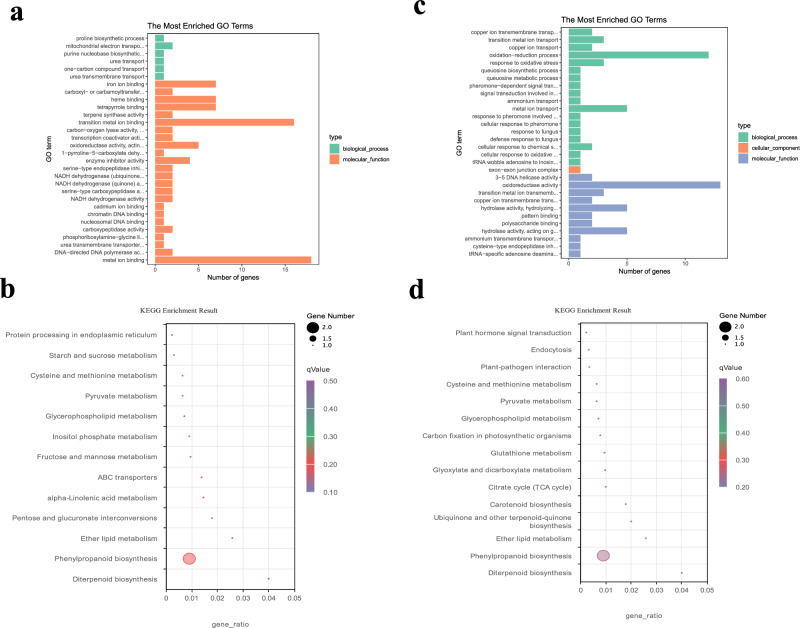
The Unigene functional enrichment result of screened DEGs. (**a**) the GO enrichment of 121 up-regulated DEGs; (**b**) the KEGG enrichment of 121 up-regulated DEGs; (**c**) the GO enrichment of 133 down-regulated DEGs; (**d**) the KEGG enrichment of 133 down-regulated DEGs.

Unigenes were processed with DESeq2 (V. 1.20.0) to acquire differential expressed genes (DEGs) with padj < 0.05 and |log2(foldchange)| > 1. The comparison of b versus a has 1379 DEGs, while 842 DEGs were down-regulated and 537 were up-regulated. The comparison of c vs. b has 3216 DEGs, while 1551 were down-regulated and 1665 were up-regulated. The comparison of c versus a has 7329 DEGs, while 3453 were down-regulated and 3876 were up-regulated. A total of 8160 non-redundant DEGs were identified. All the 8160 non-redundant DEGs were screened and filtered out 121 DEGs (Fig. S1) and 133 DEGs (Fig. S2) which increased and decreased with three bamboo developing phases respectively. The 121 up-regulated DEGs were most enriched on the GO terms of transition metal ion binding and metal ion binding, and most enriched on the pathway of Phenylpropanoid biosynthesis, and the genes of Cluster-14691.6500 and Cluster-14691.6958 play a key role in it (Fig. 3a, b). The 133 downregulated DEGs were most enriched on the GO terms of oxidation-reduction process and oxidoreductase activity, and most enriched on the pathway of Phenylpropanoid biosynthesis, and the genes of Cluster-14691.33145 and Cluster-14691.13515 play key role in it (Fig. 3c, d). The result revealed that the pathway of phenylpropanoid biosynthesis plays an important role in green bamboo shoot flavor and texture. Reverse transcription-quantitative polymerase chain reaction (RT-qPCR) assays were performed to verify the reliability of RNA-seq data (Fig. S3, Table S2).

### Metabolome basic profiles

**Figure 4:**
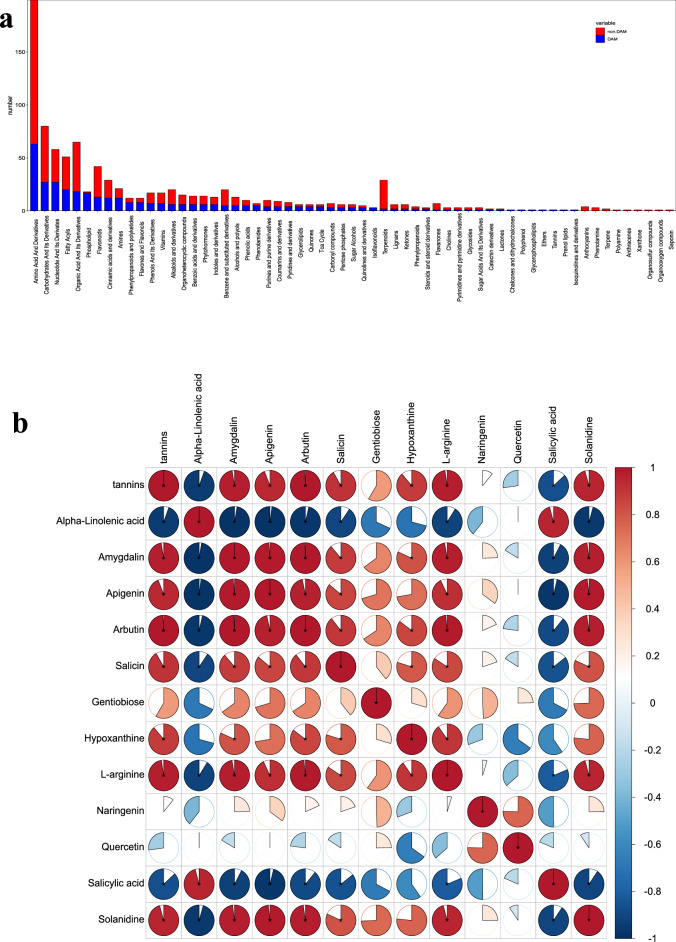
The basic profiles of the metabolome. (**a**) The statistic of DAMs and non-DAMs classes; (**b**) The correlation analysis between key bitter chemicals and tannins.

After statistics, a total of 352 non-redundant differentially accumulated metabolites (DAMs) were filtered out and classified (Fig. 4a). Of these, there are 63 kinds of metabolites belong to Amino Acid and Derivatives, 27 kinds of metabolites belong to Carbohydrates and Its Derivatives, 27 kinds of metabolites belong to Nucleotide and Its Derivates, 20 kinds of metabolites belong to Fatty Acyls, 18 kinds of metabolites belong to Organic Acid and its derivatives, 17 kinds of metabolites belong to Phospholipid, 13 kinds of metabolites belong to Flavonoids, 12 kinds of metabolites belong to Cinnamic acids and derivatives, 12 kinds of metabolites belong to Amines, 8 kinds of metabolites belong to Flavones and Flavonols, 8 kinds of metabolites belong to Phenylpropanoids and polyketides, 7 kinds of metabolites belong to Phenols and Its Derivatives, 7 kinds of metabolites belong to Vitamins (Fig. 4a).

A total of 12 non-repeated bitter chemicals were filtered out in this research. Usually, the bitter taste is companined by the astringent taste, we used the contents of tannins to compute the significant positive and negative correlated with bitter chemicals. Seven bitter chemicals (Amygdalin, Apigenin, Arbutin, Salicin, Hypoxanthine, L-arginine, and Solanidine) were significantly positively correlated with the contents of tannins. Two bitter chemicals (Alphe-Linolenic acid and Salicylic acid) were significantly negatively correlated with the contents of tannins (Fig. 4b), and several chemicals were qualified in each phase (Fig. S4). Finally, the seven positive accumulated bitter chemicals were filtered out as key bitter chemicals counting for the bitter taste transition in the shoot of *Bambusa oldhamii*.

### Association analysis between DEGs and DAMs

In the whole metabolome, 352 DAMs were identified. In comparison B versus A, 137 DAMs were identified, in which 94 DAMs significantly up accumulated including five bitter chemicals (Amygdalin, Apigenin, Arbutin, Naringenin, and Quercetin), whole 43 DAMs significantly down accumulated. All the DAMs and DEGs in this comparison were both enriched in the pathway of flavone and flavonol biosynthesis, flavonoid biosynthesis (Fig. S5). In the comparison C versus B, a total of 139 DAMs were identified, in which 50 DAMs significantly up accumulated including three bitter chemicals (Salicin, Hypoxanthine, and L-arginine), whole 89 DAMs were significantly down accumulated including one bitter chemical (Quercetin). All the DAMs in comparison C versus B. were enriched in the pathway of Glycine, serine, and threonine metabolism (Fig. S6) and all the DEGs in this comparison were enriched in the pathway of phenylpropanoid biosynthesis and plant hormone signal transduction (Fig. S6). In comparison C versus A, a total of 283 DAMs were identified, and 152 DAMs were significantly up accumulated including seven bitter chemicals (Amygdalin, Apigenin, Arbutin, Salicin, Gentiobiose, L-arginine, and Solanidine), whole 131 DAMs were significantly down accumulated including two bitter chemicals (Alpha-Linolenic acid and Salicylic acid). All the DAMs in comparison C versus A were enriched in the pathway of oxidative phosphorylation, tyrosine metabolism, and propanoate metabolism (Fig. S7), and all the DEGs in this comparison were enriched in the pathway of phenylpropanoid biosynthesis, phenylalanine metabolism, and glutathione metabolism (Fig. S7).

There are 8160 DEGs and 352 DAMs in the transcriptomic and metabolomics data respectively. The function of cor. test in the R package was used to calculate the correlation of 8160 DEGs to each DAM one by one and screened out the correlation relationship whose absolute coefficient was above 0.9 and *p* value ≤ 0.01. All the relationships merged in one file as the input file to Cytoscape software (V. 3.40) and analyzed the network (Fig. S8) (Table S3). The statistic of the correlation network was showen on (Table S3), the top 20 DAMs with degree sorted were filtered out (Arbutin, alpha-Arbutin, (2S)-2-Isopropylmalate, Melezitose, L-Alanyl-L-Phenylalanine, Methylmalonate, 5-O-p-Coumaroylquinic acid, sn-Glycero-3-phosphocholine, 9,10-EODE, L-Rhamnose, Oleamide, Succinic acid, L-Fucose, (-)-N-[3′,4′-Dihydroxy-(E)-cinnamoyl]-L-glutamic acid, Methyl linolenate, L-(+)-Rhamnose Monohydrate, L-Leucyl-L-phenylalanine, p-Hydroxybenzaldehyde, L-arginine, Amygdalin). Which, Arbutin, L-arginine, and Amygdalin were regarded as bitter chemicals.

### Pathways and transcription factors related to key bitter chemicals

All the Unigenes were annotated with iTAK (V. 1.2) software for transcription factors, 2445 non-redundant transcription factors were acquired, and 585 Transcription factors were identified as differentially expressed transcription factors (DETF). Transcription factor family of all the 585 DETF showed that 83 DETF belongs to AP2/ERF family, 19 DETF and 44 DETF belong to MYB and MYB-related family, 42 DETF belong to the NAC family, 33 DETF belong to the WRKY family, 29 DETF belong to bHLH family, 28 DETF belong to HB family, 23 DETF belong to C2H2 family, and 22 DETF belong to TCP family (Table S4). The correlation analysis between these seven key bitter chemicals accumulation patterns with the 585 DETFs expression pattern was analyzed. The absolute value of a correlation coefficient above 0.9 was filtered out and shown in a Chord Diagram.

**Figure 5:**
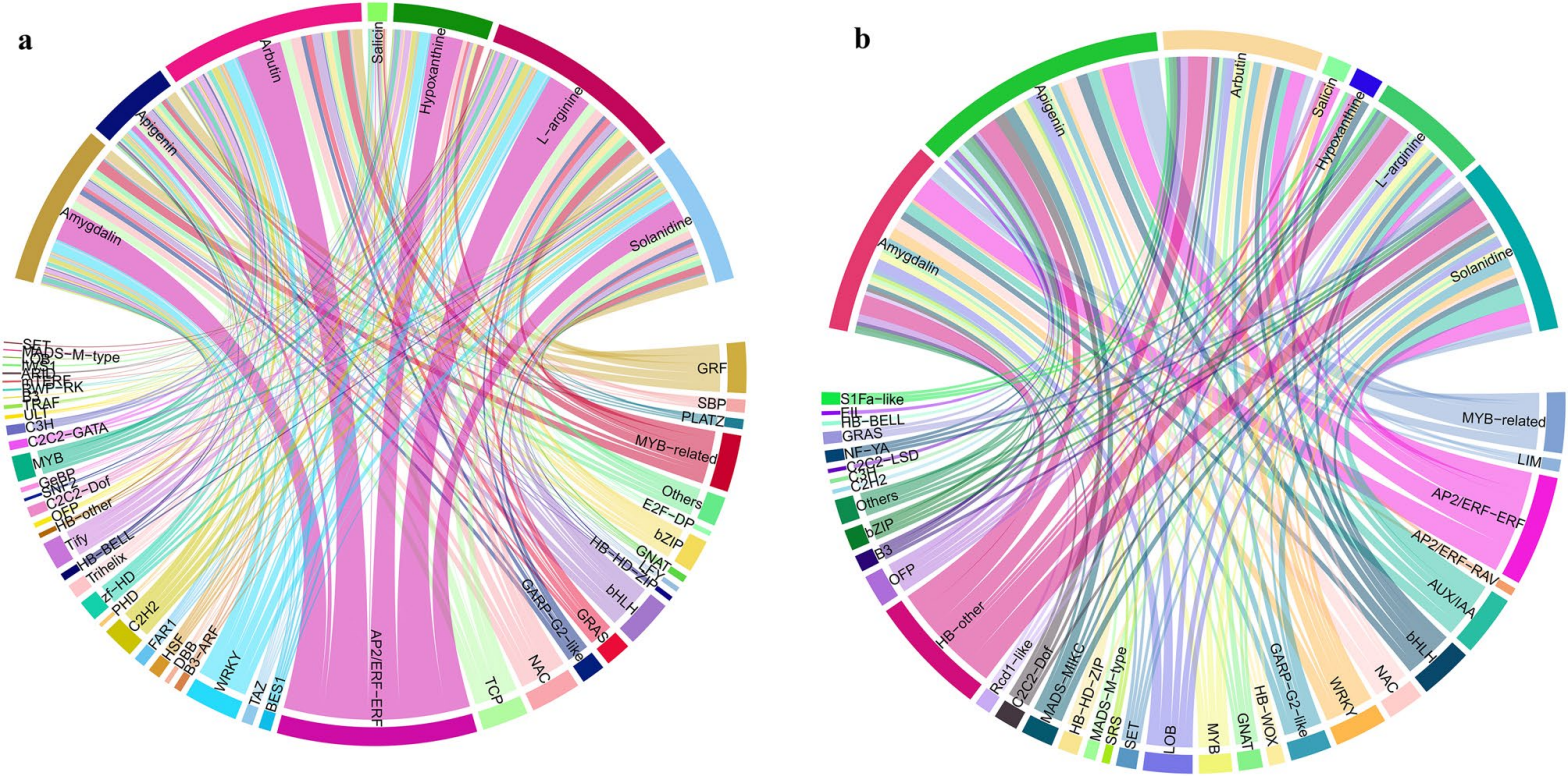
The correlated relationship between key bitter chemicals and DETFs. (**a**) The positively correlated relationships between the key bitter chemicals and DETFs; (**b**) The negatively correlated relationships between the key bitter chemicals and DETFs.

The number of DETFs positively correlated to Amygdalin, Apigenin, Arbutin, Salicin, Hypoxanthine, L-arginine, and Solanidine is 170, 90, 213, 21, 104, 209, and 151 respectively. A total of 958 positive correlated relationships between the seven bitter chemicals and DETFs, and those relationships were shown on the ChordDiagram (Fig. 5a). The results showed that AP2/ERF-ERF might be the major transcription factor family positively regulates the key bitter chemicals accumulation. The number of DETFs negatively correlated to Amygdalin, Apigenin, Arbutin, Salicin, Hypoxanthine, L-arginine, and Solanidine is 49, 63, 39, 6, 7, 28, and 44 respectively. A total of 236 negative correlated relationships between the seven bitter chemicals and DETFs, and those relationships were shown on the ChordDiagram (Fig. 5b). The results showed that AP2/ERF-ERF might be the key transcription factor families that negatively regulated the key bitter chemicals accumulation. A total of 260 non-repeated DETFs were screened out from the 958 positive correlated relationships, and a total of 91 non-repeated DETFs were screened out from the 236 negative correlated relationships. The family distribution of all the non-repeated DETFs was shown on the bar chart (Fig. S9).

## Discussion

The content accumulation of bitter chemicals (Alpha-Linolenic acid and Salicylic acid) was identified as decreased accumulation with the three green bamboo shoots’ development phases. Alpha-Linolenic acid (ALA) is an 18-carbon polyunsaturated omega-3 fatty acid, and is an essential component of nutrition for all mammals, coming from many common vegetable oils. Alpha-Linolenic acid (ALA) could be used as a nutraceutical and pharmaceutical and has been reported to have cardiovascular-protective, anti-cancer, neuro-protective, anti-osteoporotic, anti-inflammatory, and antioxidative effects^16,17^. Salicylic acid is a plant defense hormone produced from chorismate via two independent pathways through isochorismate synthase (ICS) and phenylalanine ammonia lyase (PAL), and plays many biological processes in the plant, like growth, flowering, redox regulation, and apical hoot formation^18^. The transcription factor family of WRKY and MYB participate in the SA biosynthesis and metabolism pathway regulation^18,19^.

Bitter chemicals like Solanidine, Amygdalin, Arbutin, Apigenin, Salicin, and others were identified in the bamboo shoots whose contents were increased with three bamboo shoot developing stages. Solanidine is a steroidal alkaloid derived from cholesterol, extracted from Solanaceous plants, such as potatoes (*Solanum tuberosum*). In potatoes, the steroidal glycoalkaloid biosynthetic pathway has been reported, SGT1 (solanidine galactosyltransferase) and SGT2 (solanidine glucosyltransferase) initiate the glycosylation of solanidine to γ-solanine and γ-chaconine, respectively, and SGT3 (rhamnosyltransferase) catalyzes the conversion to α-solanine and α-chaconine^20^. The biosynthesis of solanidine has also been summarized in the Biosynthesis of various alkaloids (https://www.kegg.jp/pathway/map00996) and Biosynthesis of alkaloids derived from terpenoid and polyketide (https://www.kegg.jp/pathway/map01066). Amygdalin has pharmacological activities of anti-tumor, anti-fibrotic, anti-inflammatory, analgesic, immunomodulatory, anti-atherosclerosis, ameliorating digestive system and reproductive system, and also exist some toxicity properties^21^. Amygdalin comes from a series of enzymes with Phenylalanine^22^ shown in Cyanoamina acid metabolism (https://www.kegg.jp/pathway/map00460). Arbutin has the properties of antibacterial activity, urinary tract infections, anti-melanogenic activity, antioxidant, anti-inflammatory and antitumor activities^23^ and participates in the pathway of Glycolysis/Gluconeogenesis (map00010) and Phosphotransferase system (PTS) (map02060). Apigenin is an anticancer agent, that could prevent atherogenesis, hypertension, cardiac hypertrophy, autoimmune myocarditis, and other therapeutic potentials^24^, and play roles in the pathway of Flavonoid biosynthesis (map00941), Isoflavonoid biosynthesis (map00943), Flavone and flavonol biosynthesis (map00944), and Biosynthesis of phenylpropanoids (map01061).

Salicin, a type of phenolic glycoside, extracted from willow bark, especially in the phloem^25^, is a substance of aspirin. Phenolic glycosides are products of phenylalanine and tyrosine metabolism^26^. In mammals, the bitter taste receptor is usually conducted by the family of TAS2Rs genes^27^, seven transmembrane G protein-coupled receptors usually expressed in taste bud cells ^27^. The treatment of salicin and MeJA could induce the expression of WRKY transcription factors in *Withania somnifera*, such as WsWRKY1^28^.

The APETALA2/ETHYLENE RESPONSE FACTOR (AP2/ERF) gene could modulate diverse metabolites biosynthesis, such as steroidal glycoalkaloids, nicotine, and others^29^. Chalcone isomerase (CHI) family proteins play roles in flavonoid biosynthesis which was regulated by AP2/ERF family^3^. Flavonoids were one of the dietary phytonutrients which were composed of diverse bioactive compounds^1^. The flavonoid biosynthesis is controlled by multiple transcription factors (TFs) such as WD40 repeat, bHLH, MYB, and others. The bitterness in melon was caused by the accumulation of cucurbitacin B, and the genes in Cu B biosynthetic pathway were regulated by bHLH transcription factors such as CmBr (Bitter root) and CmBt (Bitter fruit)^5^. The R2R3-MYB transcription factor could regulate the gene expression in the flavonoid pathway^6^. The phenols, flavonoids, isoflavones, and others were always bitter, acrid, and astringent^1^.

The bamboo shoot of *Bambusa oldhamii* Munro has a great ecomomic value in the market because of its deliciously sweet, crispy, and tender taste^30^. Besides regard as fresh vegetables, bamboo food can process into manufactured foods such as bamboo shoot juice, boiled bamboo shoot can, dried bamboo shoots, chutney, and pickles^29^. If the bamboo shoot was dug out from under ground in the morning, the price was higher than those stretched out of the soil surface with several days. And the taste changed to a litter bitterness when the shoot grew out from underground to aboveground with several days of sunlight. From a previous study, cyanogenic glycosides is a potential toxic and bitter compound in bamboo shoots, which would produce cynahydric acid whose contents could be removed from boiling, canning, soaking, and fermentation^31^. Other researchers supported that L-phenylalanine, uridine, L-tryptophan, and adenine caused bamboo shoot bitterness, and L-phenylalanine being the greatest contributor^12^. In this research, the bitter chemicals of Solanidine, Amygdalin, Arbutin, Apigenin, Salicin, and others would count for the bitter taste formation from underground to above ground of *Bambusa oldhamii*. And the AP2/ERF-ERF family might participate in the taste transition process. This is a basic research, more developing phases, new detection methods, and widely further research was needed for future mining bamboo shoot bitter chemicals and flavor transition. The taste and flavor research of bamboo shoots would lay foundation for bamboo breeding and taste improvement.

## Methods

### Plant materials

The bamboo shoots of *Bambusa oldhamii* Munro were picked in the bamboo garden located in Wenzhou City (Zhejiang, China) in the summer. Usually, the delicious tasty edible green bamboo shoot is the middle part, when the bamboo is growing and stretched out from the ground, the top part has a little bitter tasty when explored to sunlight for days, with time growing and more sunlight accepted, the edible bamboo shoots would be grown and differentiated into inedible tender culms. To research the bitterness chemicals and formation when green bamboo grows from underground with darkness to aboveground with sunlight, the top part of green bamboo with three developing stages were chosen to research those questions. Three developing phases were picked for searching the chemical compounds and formation mechanism of bitterness tasty when the bamboo shoots grow aboveground and are exposed to sunlight. When the bamboo shoot grown in underground and the appearance of the shoot sheath was etiolated, the culm differentiation tissues of the cross section of this period shoot wasn’t visible, we regard this period as phase 1 (Fig. 1a). Phase 1 (Fig. 1a) includes three biological replicates samples marked A1–A3 in the metabolome and a1-a3 in the transcriptome. When the shoot apex has just touched the ground surface and emerged, the override soil of the bamboo shoot became loose, and the foliole of the shoot sheath has stretched out but is still etiolated, the culm differentiation tissues of the cross section of the shoot would be visible as milky white circle tissues, this period regard as phase 2 (Fig. 1b). Phase 2 (Fig. 1b) includes three biological replicates samples marked B1–B3 in the metabolome and b1–b3 in the transcriptome.

The shoot apex has emerged from the ground surface about 5-8cm, and the foliole of shoots sheath have become green under light, the culm differentiation tissues of the cross section of the shoot has elongated and visible clearly, this period was regarded as phase 3 (Fig. 1c). Phase 3 (Fig. 1c) includes three biological replicates samples marked C1–C3 in the metabolome and c1–c3 in the transcriptome. The sample tissues were cut from top to middle (about 5 cm length of shoot tip tissues) after stripping sheaths of bamboo shoots. The cut bamboo shoots slices are frozen into liquid nitrogen and ground into powder, and stored in an ultra-low temperature freezer at − 80 °C for RNA and metabolite extraction.

### Physicochemical property detection

The detection kit of lignin (Item no. BC4205), cellulose (Item no. BC4285), and hemicellulose (Item no. BC4445) contents were purchased from Beijing Solarbio Science & Technology Co., Ltd. The detection kit of tannin contents (Item no. JC0106-M) was purchased from Genepioneer Biotechnologies. The contents of flavone were detected with public plant flavone detection methods (DB43/T 476-2009). The insoluble protein contents detection was used with traditional Coomassie brilliant blue methods. The One-way ANOVA method with Multiple comparisons test (Tukey) was used for statistical analysis.

### RNA library construction and sequencing

The RNA library and sequencing were processed in Novogene Co., Ltd. Total RNA was extracted with Plant Total RNA Kit (QIAGEN, Germany). Total amounts and integrity of RNA were assessed using the RNA Nano 6000 Assay Kit of the Bioanalyzer 2100 system (Agilent Technologies, CA, USA). Total RNA was used as input material for the RNA sample preparations, and mRNA was purified using poly-T oligo-attached magnetic beads. After the first and second-strand cDNA was synthesized, DNA fragments were ligated with adaptors and purified with the AMPure XP system (Beckman Coulter, Beverly, USA). After PCR amplification, the PCR product was purified by AMPure XP beads, and the library was finally obtained and quantified by Qubit2.0 Fluorometer and Agilent 2100 bioanalyzer to ensure the quality of the library. The library is qualified and sequenced by the Illumina NovaSeq 6000 (Illumina, USA). Raw data (raw reads) of fastq format were first processed through in-house Perl scripts. In this step, clean data (clean reads) were obtained by removing reads containing adapter, reads containing N base and low-quality reads from raw data. At the same time, Q20, Q30, and GC contents of the clean data were calculated. All the downstream analyses were based on clean data with high quality.

### Transcriptome property analysis

After the clean reads were obtained, the Trinity software^32^ (V. 2.6.6) (min_kmer_cov set to 2, other parameters set as default) was used to assemble the clean reads to transcripts. The software of Corset^33^ (V. 4.6) aggregated transcripts into many clusters according to inter-transcriptional Shared Reads, each cluster was defined as “Gene”. Coding sequence (CDS) information was predicted with NR and Swissport protein library, and the sequences without predicted results were predicted by TransDecoder (3.0.1) software. Genes were annotated in seven databases, Nr (NCBI non-redundant protein sequences); Nt (NCBI non-redundant nucleotide sequences); Pfam (Protein family); KOG/COG (clusters of Orthologous Groups of proteins); Swiss-Prot (A manually annotated and reviewed protein sequence database); KO (KEGG Ortholog database); GO (Gene Ontology). The plant transcription factors were predicted with iTAK software.

Gene expression was evaluated with FPKM^34^ (expected number of Fragments Per Kilobase of transcript sequence per Millions base pairs sequenced). Differential expression analysis was performed using the DESeq2 R package (V. 1.20.0). The resulting P-values were adjusted using Benjamini and Hochberg’s approach for controlling the false discovery rate (FDR). Padj < 0.05 and |log2(fold change)|> 1 were set as the threshold for significantly differential expression. GOseq^35^ (1.10.0) and KOBAS (V2.0.12) software were used for GO function enrichment analysis and KEGG pathway enrichment analysis of differential gene sets. The GO and KEGG enrichment of filtered DEGs were analyzed and drawn with ClusterProfiler^36^, and the heatmap was drawn with the Pheatmap package in R software. The Validation process of RNA-seq data was samed with our previous reported methods, but performed on QuantStudio 7 Flex Real-Time PCR System^37^.

### Metabolites extraction and measurements

The top part of bamboo shoots (100 mg) was individually grounded with liquid nitrogen and the homogenate was resuspended with prechilled 80% methanol by the well vortex. The samples were incubated on ice for 5 min and then centrifuged at 15,000*g*, 4 °C for 20 min. Some of the supernatants were diluted to a final concentration containing 53% methanol by LC–MS grade water. The samples were subsequently transferred to a fresh Eppendorf tube and then were centrifuged at 15,000*g*, 4 °C for 20 min. Finally, the supernatant was injected into the LC–MS/MS system analysis. The metabolic extraction and purification procedures followed those of a previous study, and the conditions and parameters for LC–MS/MS assays were similar to those used in a previous study^38^.

### HPLC–MS/MS for metabolome

The metabolic extraction and purification procedures followed those of a previous study, and the conditions and parameters for LC–MS/MS assays were similar to those used in a previous study^39^. LC–MS/MS analyses were performed using an ExionLC™ AD system (SCIEX) coupled with a QTRAP^®^ 6500+ mass spectrometer (SCIEX) in Novogene Co., Ltd. (Beijing, China). Samples were injected onto a Xselect HSS T3 (2.1** × **150 mm, 2.5 μm) using a 20 min linear gradient at a flow rate of 0.4 mL/min for the positive/negative polarity mode. The eluents were eluent A (0.1% Formic acid–water) and eluent B (0.1% Formic acid-acetonitrile). The solvent gradient was set as follows: 2% B, 2 min; 2–100% B, 15.0 min; 100% B, 17.0 min; 100–2% B, 17.1 min; 2% B, 20 min. QTRAP^®^ 6500 + mass spectrometer was operated in positive polarity mode with Curtain Gas of 35 psi, Collision Gas of Medium, IonSpray Voltage of 5500 V, Temperature of 550 °C, Ion Source Gas of 1:60, Ion Source Gas of 2:60. QTRAP^®^ 6500+ mass spectrometer was operated in negative polarity mode with Curtain Gas of 35 psi, Collision Gas of Medium, IonSpray Voltage of − 4500 V, Temperature of 550 °C, Ion Source Gas of 1:60, Ion Source Gas of 2:60.

### Metabolites identification and analysis

The detection of the experimental samples using MRM (Multiple Reaction Monitoring) was based on the novogene in-house database. The Q3 was used for the metabolite quantification. The Q1, Q3, RT (retention time), DP (declustering potential), and CE (collision energy) were used for the metabolite identification. The data files generated by HPLC–MS/MS were processed using the SCIEX OX (Version 1.4) to integrate and correct the peak. The main parameters were set as follows: minimum peak height, 500; signal/noise ratio, 5; gaussian smooth width, 1. The area of each peak represents the relative content of the corresponding substance.

These metabolites were annotated using the Kyoto Encyclopedia of Genes and Genomes (KEGG), Human Metabolome Database (HMDB), and LIPID MAPS database. We applied univariate analysis (t-test) to calculate the statistical significance (*P* value). The metabolites with VIP > 1 and *P* value < 0.05 and fold change ≥ 2 of FC ≤ 0.5 were considered as DAMs. The metabolic pathways enrichment of differential metabolites was performed, when the P-value of metabolic pathway < 0.05, metabolic pathways were considered as enrichment. Cluster trends of differential metabolites were plotted by the Mfuzz package in R language. The correlation analysis of differential genes and differential metabolites is based on Pearson statistical method in the R language mixOmics package to calculate the correlation coefficient r^2^ and the *P* value of differential genes and differential metabolites. The chord diagram was created in R using the circlize package^40^.

### The detection of Vitamin B2

The tissues (0.2 g) with 1 Ml 0.05 mol/L sodium acetate water solution and homogenate in ice bath condition and incubated 30 min. After centrifuging (8000*g*, 10 min), the supernatant liquid was filtered with filtrator, and stored in low temperature and darkness environments until HPLC detection. RGOL L-3000 HPLC System with Sepax Bio-C18 (250 mm × 4.6 mm,5 μm) at 35 °C was employed for the detection. The flow rate was 1 mL/min, and the injection volume was 10 μL, and detected at 270 wave length.

### The detection of arbutin, Salicylic acid and Salicin

Tissues were grounded with liquid nitrogen, and 500 mg tissue powders was suspended with 4 ml 1% hydrochloric acid—methanol solution and by well vortex, incubated 30 min in ice condition; 11000 rpm, 30 min, final supernatant liquid up to 10 mL, mixing and filtering with 0.22 μm organic phase filterable membrane. Then detected with an Agilent 1260 Infinity II (Agilent Technologies, Germany) coupled with an Agilent Technologies 6420 Triple Quad LC/MS system. Agilent ZORBAX Eclipse Plus C18 (3.5 μm, 2.1 × 150 mm) operated at 35 °C was employed. The flow rate was 0.3 mL/min, and the injection volume was 3 μL. The mobile phases were 0.1% formic aqueous solution (A) and acetonitrile (B). The gradient program [time (min), %B] was: (0.00, 20); (1.00, 90); (5.00, 90); (5.10, 20); (10.00, 20). Data were collected in the positive ESI mode separate runs on Agilent Technologies 6420 Triple Quad LC/MS system with MRM scan model. The capillary voltage was 4000 V with Gas Temp was 350 °C, Gas Flow was 10 l/min, and Nebulizer was 45 psi.

### Plant materials statement

The bamboo shoots of *Bambusa oldhamii* Munro were collected from the locally planted green bamboo forest which have acquired the permission by local planter in Wenzhou City (Zhejiang, China).

### Methods statement

All methods were carried out in accordance with relevant guidelines.

## Conclusions

Metabolome and transcriptome technologies were used to research the bitter chemicals of the green bamboo shoot when growing from underground to aboveground. A total of 8160 non-redundant DEGs, 352 DAMs, and 585 DETFs were identified in our research. A total of several bitter chemicals was finally mined out from our data, of which, the content of Solanidine, Amygdalin, Salicin, Arbutin, and others increased with the shoot-developing phases indicating these chemicals were participate in bitterness taste formation in green bamboo shoots. Correlation analysis with the transcription factors, results showed that the family of AP2/ERF TFs might play important roles in the shoot flavor changes. Combined with the KEGG pathway presentation, the result showed that the Biosynthesis of phenylpropanoids might participate in the green bamboo shoot bitter taste transition. Our study provides key bitter chemicals, useful transcription factors, and several pathways for searching for green bamboo shoot flavor and tasty transition from underground to aboveground.

## Data availability

All the raw data has been submitted to the Sequence Read Archive (SRA) of the National Center for Biotechnology Information (NCBI) with BioProject ID of PRJNA826699. The accessions of a1 are BioSample (SAMN27578175) and SRA (SRR18749978). The accessions of a2 are BioSample (SAMN27578176) and SRA (SRR18749977). The accessions of a3 are BioSample (SAMN27578177) and SRA (SRR18749976). The accessions of b1 are BioSample (SAMN27578178) and SRA (SRR18749975). The accessions of b2 are BioSample (SAMN27578179) and SRA (SRR18749974). The accessions of b3 are BioSample (SAMN27578180) and SRA (SRR18749973). The accessions of c1 are BioSample (SAMN27578181) and SRA (SRR18749972). The accessions of c2 are BioSample (SAMN27578182) and SRA (SRR18749971). The accessions of c3 are BioSample (SAMN27578183) and SRA (SRR18749970). This Transcriptome Shotgun Assembly project has been deposited at DDBJ/EMBL/GenBank under the accession GJWP00000000. The version described in this paper is the first version, GJWP01000000.

### Supplementary Information

Supplementary Legends.

Supplementary Figure S1.

Supplementary Figure S2.

Supplementary Figure S3.

Supplementary Figure S4.

Supplementary Figure S5.

Supplementary Figure S6.

Supplementary Figure S7.

Supplementary Figure S8.

Supplementary Figure S9.

Supplementary Table S1.

Supplementary Table S2.

Supplementary Table S3.

Supplementary Table S4.
