# Supplementary material for: Transcriptomic and metabolomic analyses reveal the flavor of bitterness in the tip shoots of Bambusa oldhamii Munro
Source: Sci Rep. 2023 Sep 8;13:14853. doi: 10.1038/s41598-023-40918-8 (PMC10491673; doi:10.1038/s41598-023-40918-8)
Supplement: Supplementary file 1 — Supplementary Legends. [file 41598_2023_40918_MOESM1_ESM.docx]

**Supplementary Figure legend:**

**Fig. S1**: The expression pattern of 121 up-regulated DEGs.

**Fig. S2**: The expression pattern of 133 down-regulated DEGs.

**Fig. S3**: The qPCR validations of four putative genes in the RNA-seq data. The bar charts show the qPCR results in three green bamboo shoot phases; the line charts show the FPKM values of these unigenes. The qPCR results represent the mean (±SD) of three biological replicates.

**Fig. S4**: The verification of four metabolites in the metabolome data. The bar chart represents the mean (±SD) of three technique replicates with mixed samples in each phase.

**Fig. S5**: The KEGG enrichment of all the DEGs and DAMS in comparison B vs. A

**Fig. S6**: The KEGG enrichment of all the DEGs and DAMS in comparison C vs B

**Fig. S7**: The KEGG enrichment of all the DEGs and DAMS in comparison C vs. A

**Fig. S8**: The undirected correlation network of all DEGs (big circle) and all DAMs (small circle).

**Fig. S9**: The family distribution of all the non-repeated DETFs

**Supplementary Table legend:**

**Table S1**: The summary of RNA-seq data quality.

**Table S2**: The primers used for qPCR in this search.

**Table S3**: The node attributes of the correlation network between all DEGs and all DAMs

**Table S4**: The identified family of DETFs in this research.
