# Supplementary figures and images for: Transcriptomic and metabolomic analyses reveal the flavor of bitterness in the tip shoots of Bambusa oldhamii Munro
Source: Sci Rep. 2023 Sep 8;13:14853. doi: 10.1038/s41598-023-40918-8 (PMC10491673; doi:10.1038/s41598-023-40918-8)

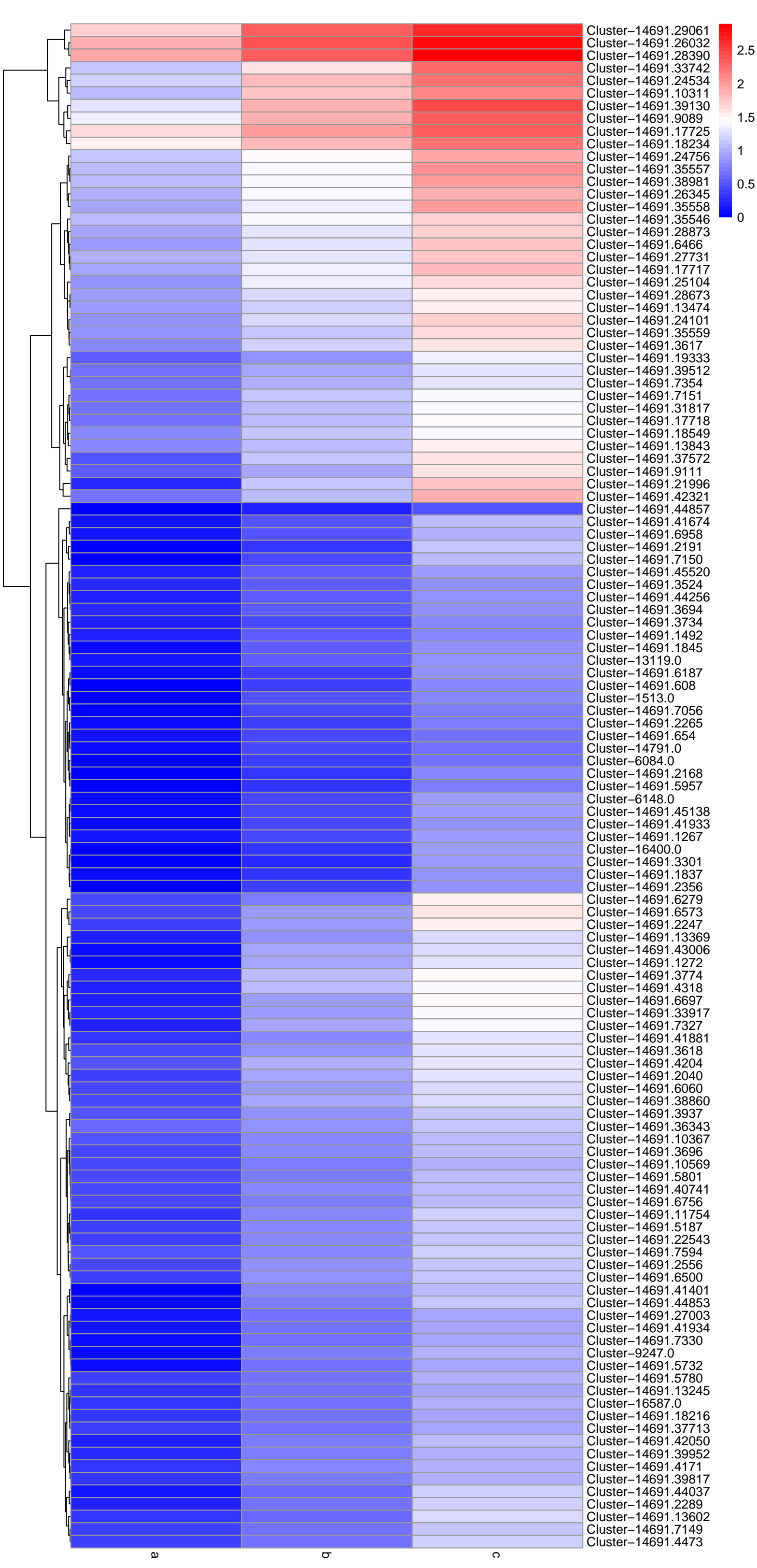

Supplement: Supplementary file 2 — Supplementary Figure S1. [file 41598_2023_40918_MOESM2_ESM.pdf]

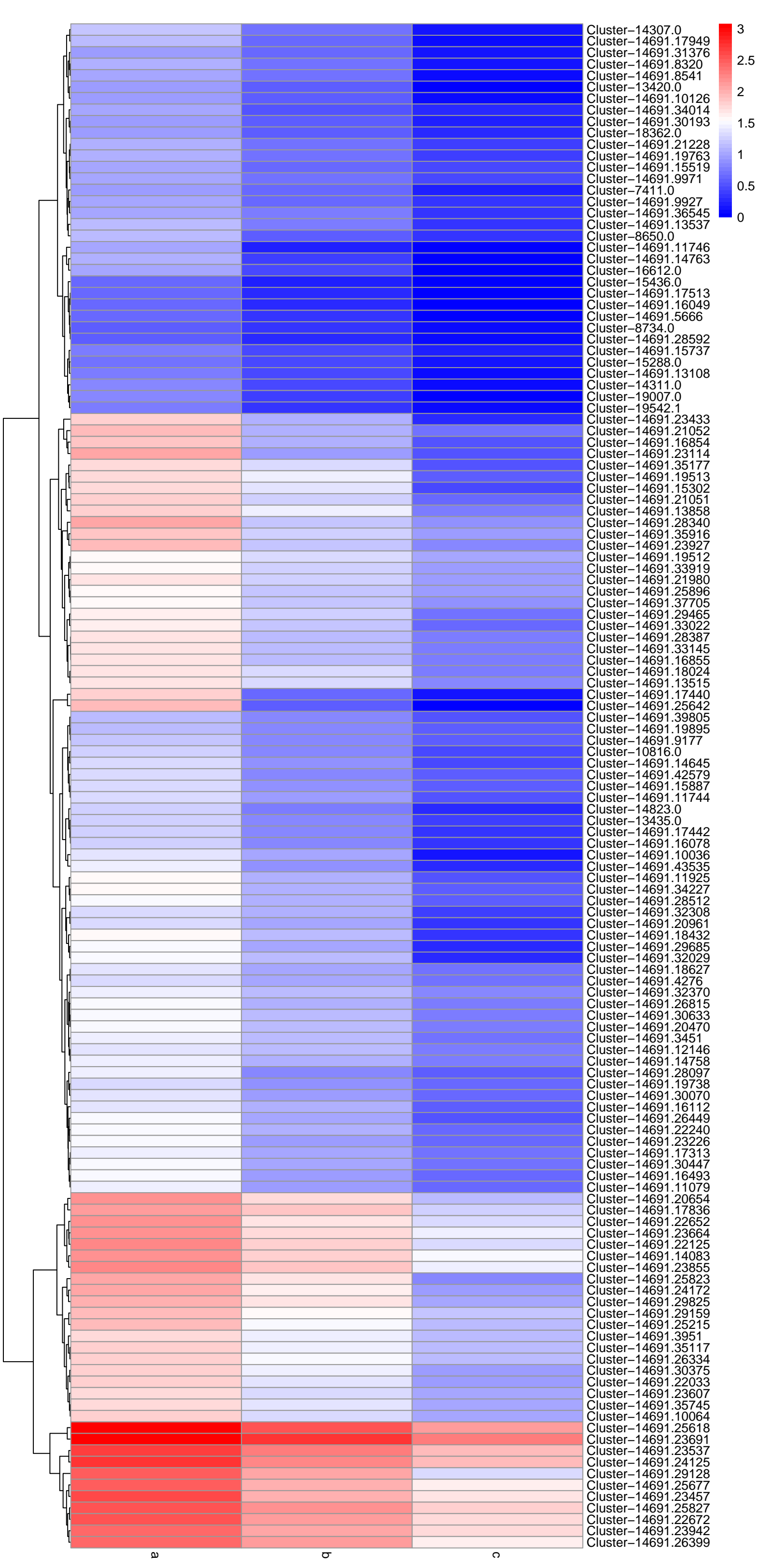

Supplement: Supplementary file 3 — Supplementary Figure S2. [file 41598_2023_40918_MOESM3_ESM.pdf]

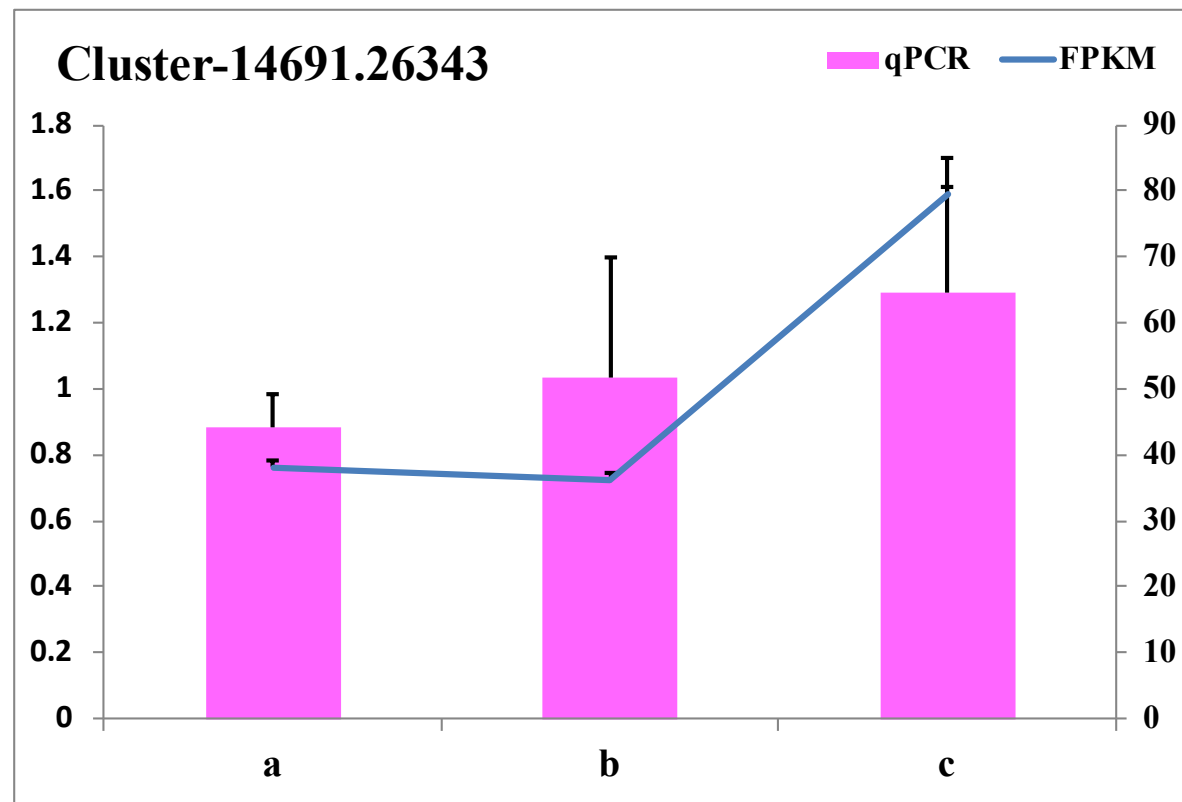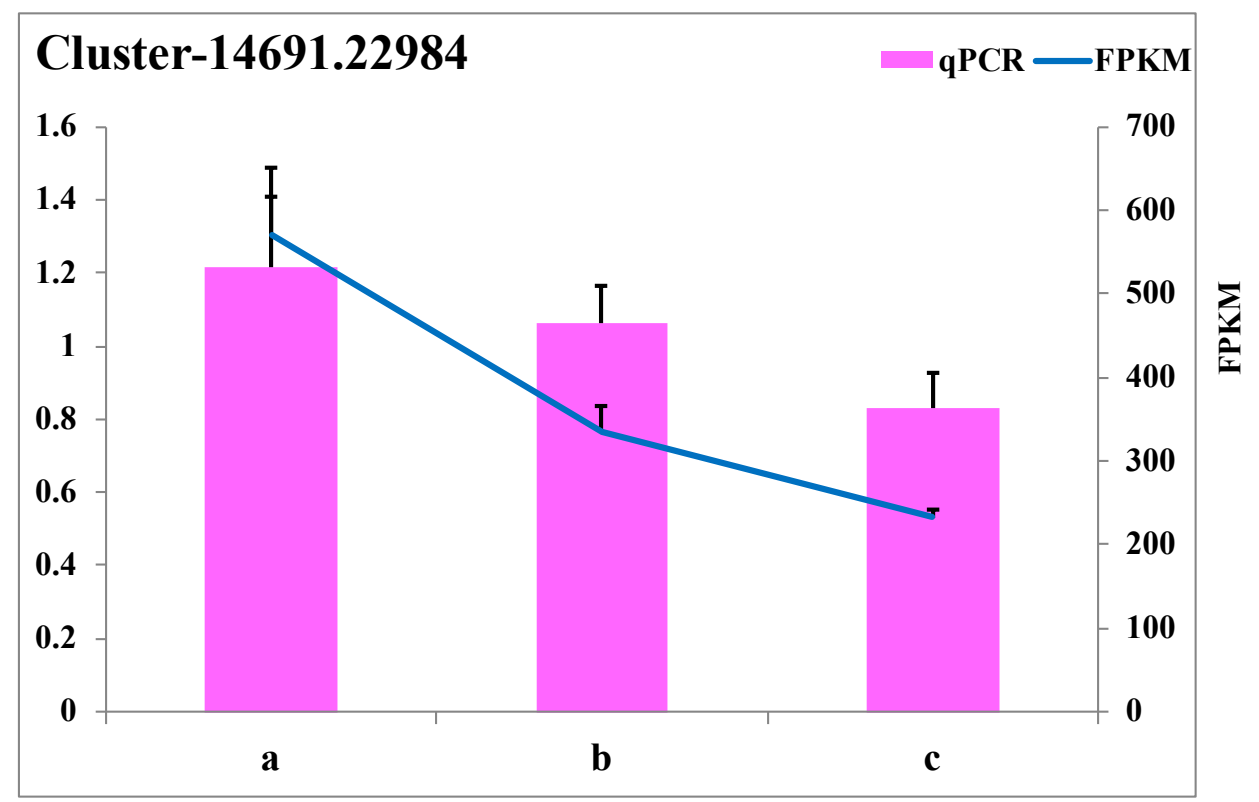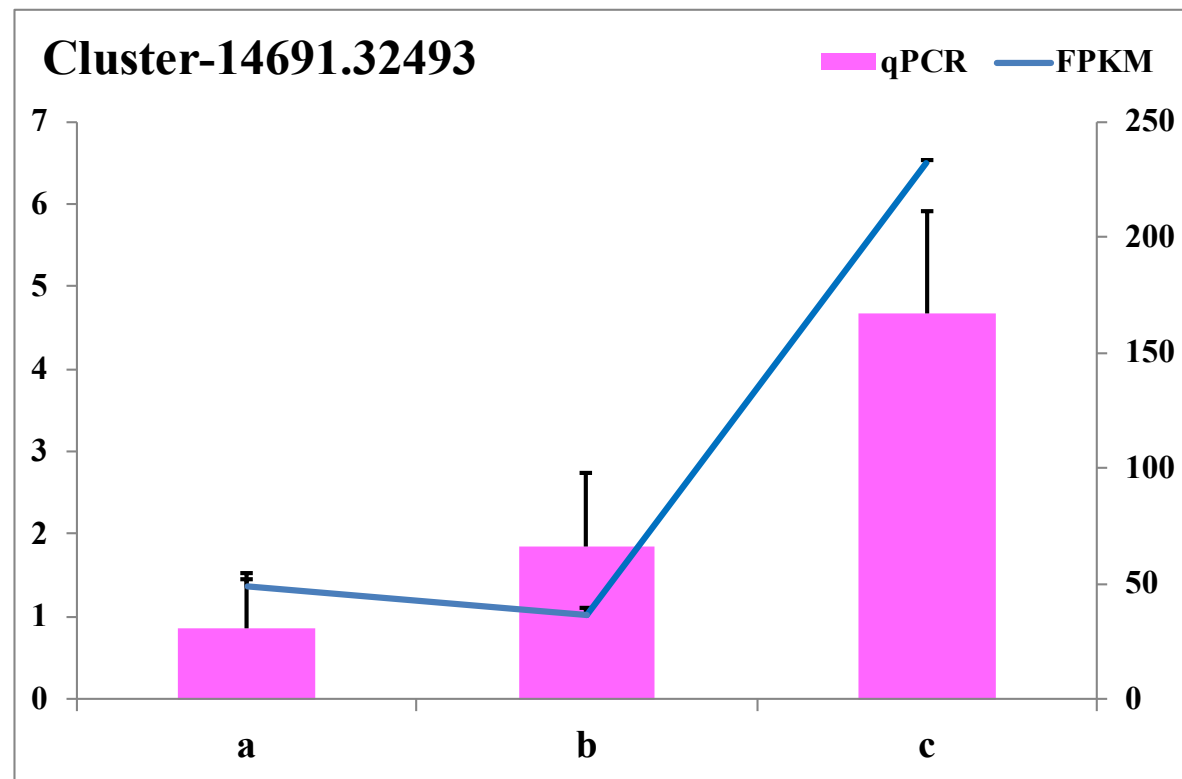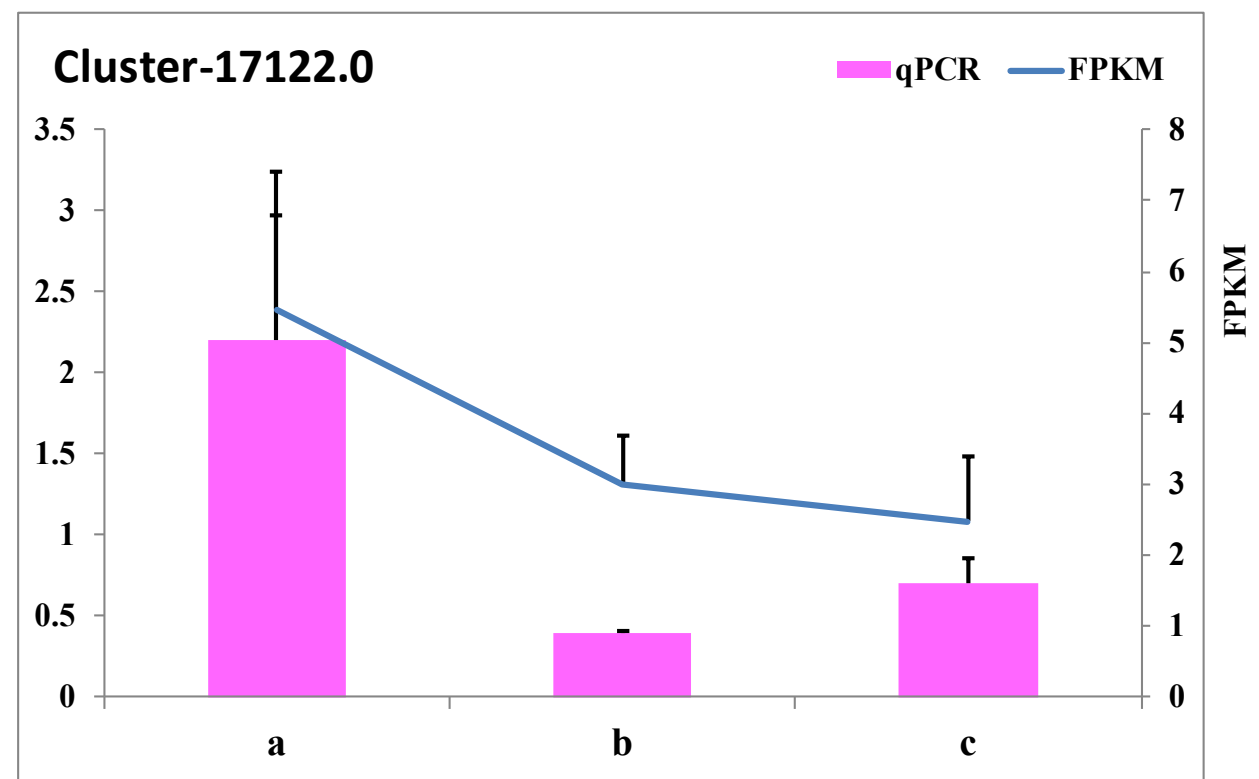

Supplement: Supplementary file 4 — Supplementary Figure S3. [file 41598_2023_40918_MOESM4_ESM.pdf]

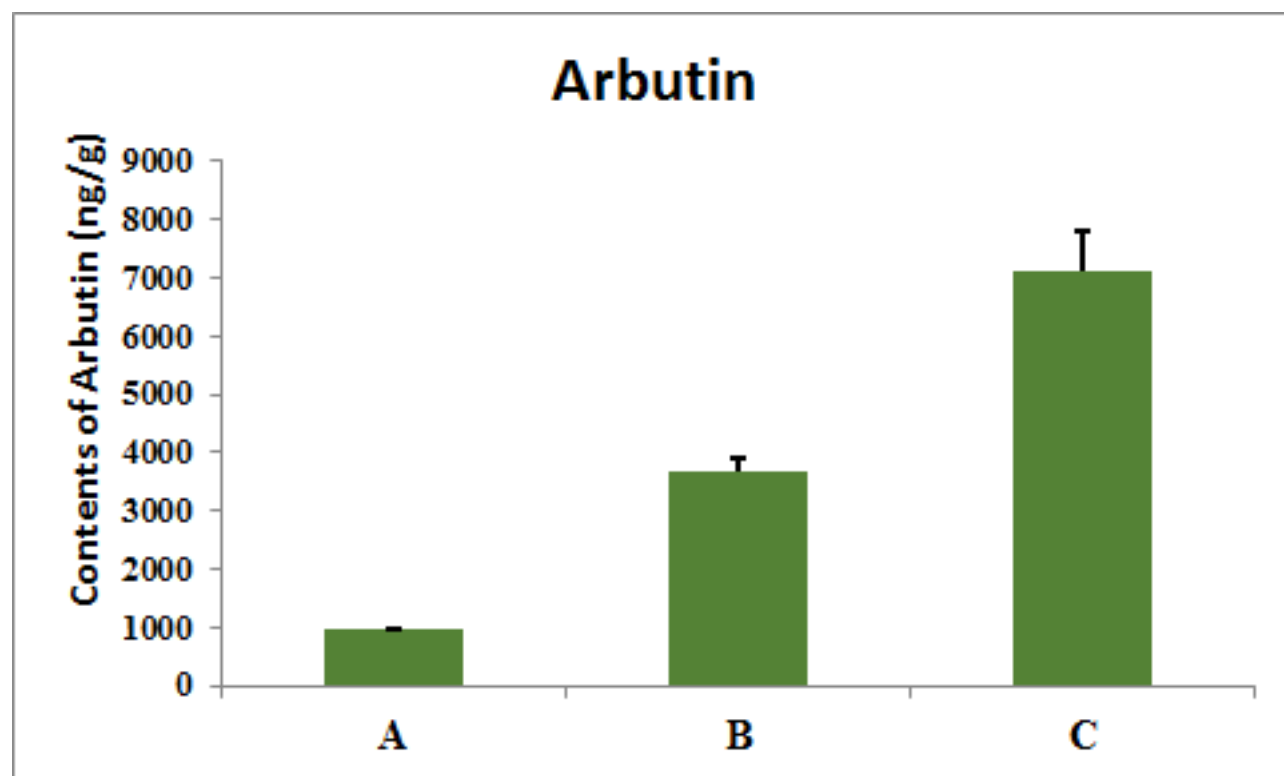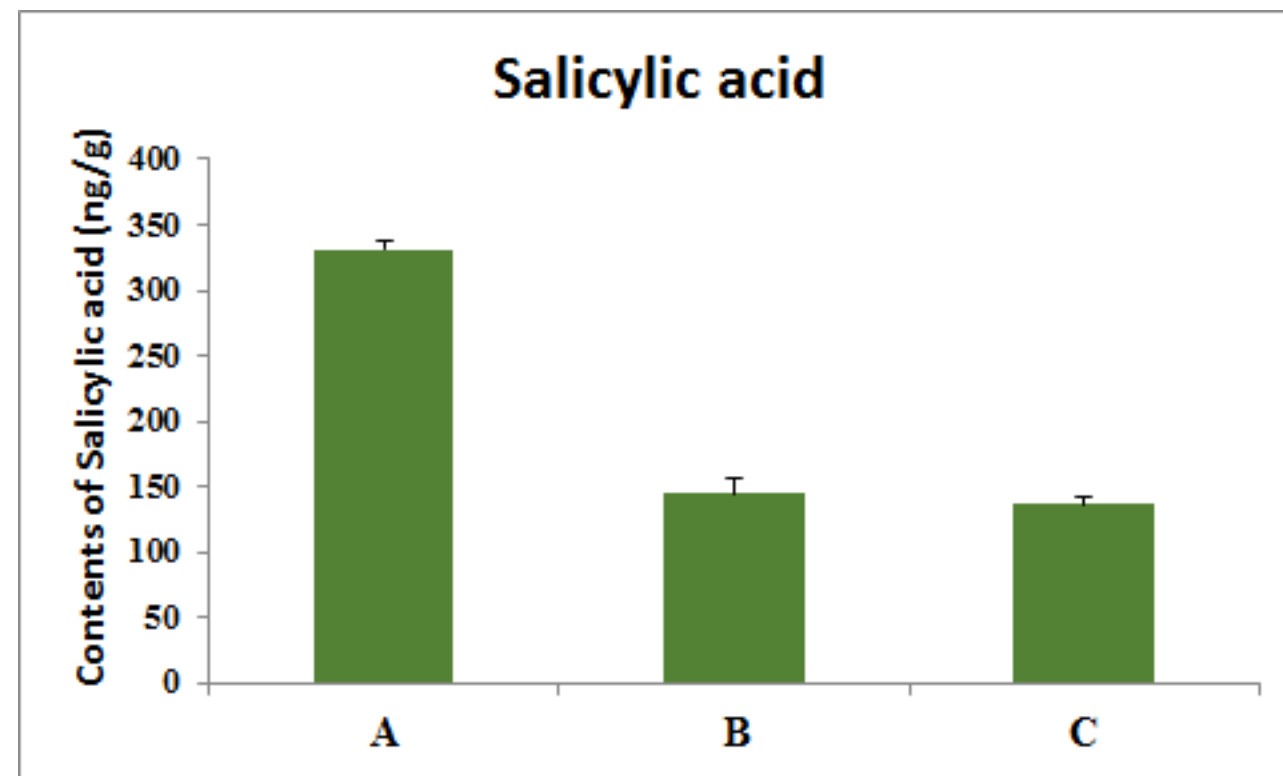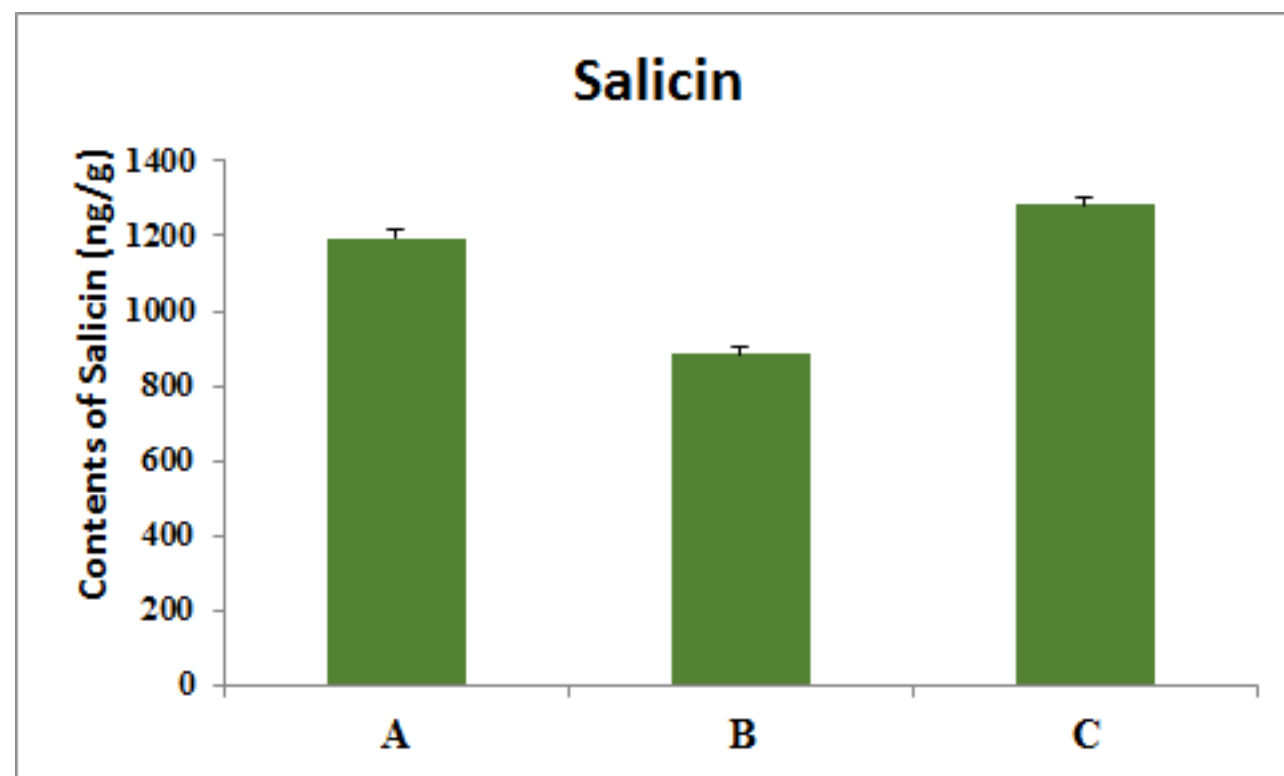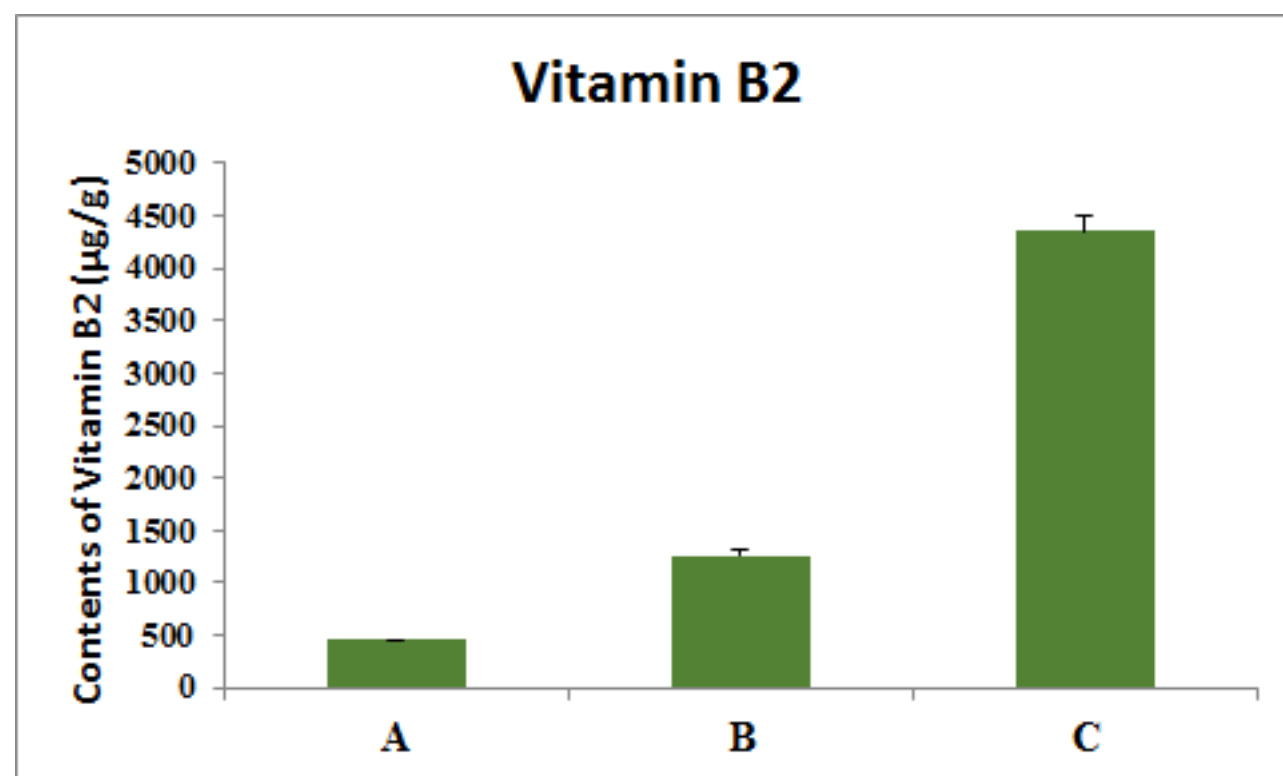

Supplement: Supplementary file 5 — Supplementary Figure S4. [file 41598_2023_40918_MOESM5_ESM.pdf]

# B.vs.A

kegg description

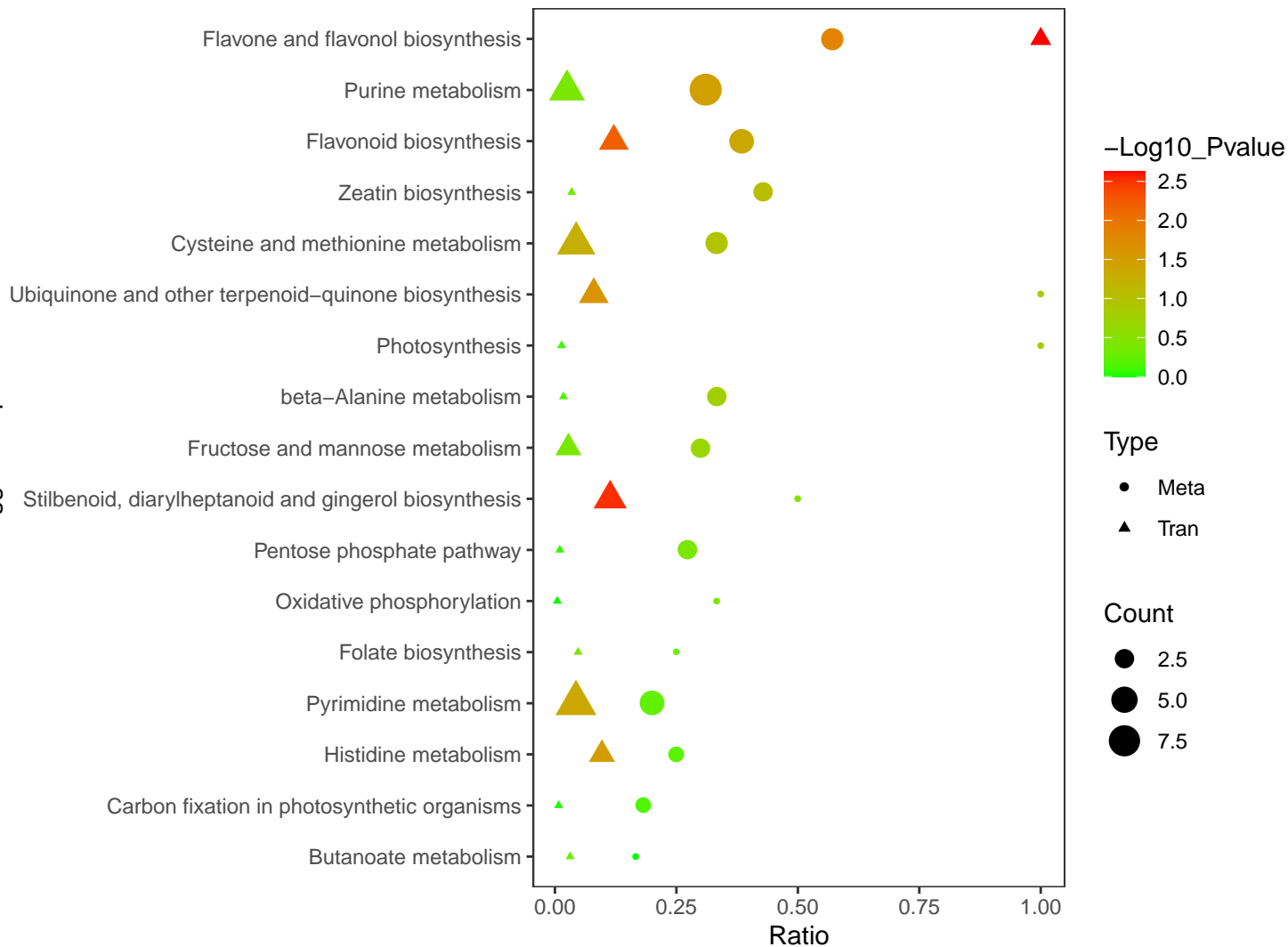

Supplement: Supplementary file 6 — Supplementary Figure S5. [file 41598_2023_40918_MOESM6_ESM.pdf]

C.vs.B

kegg description

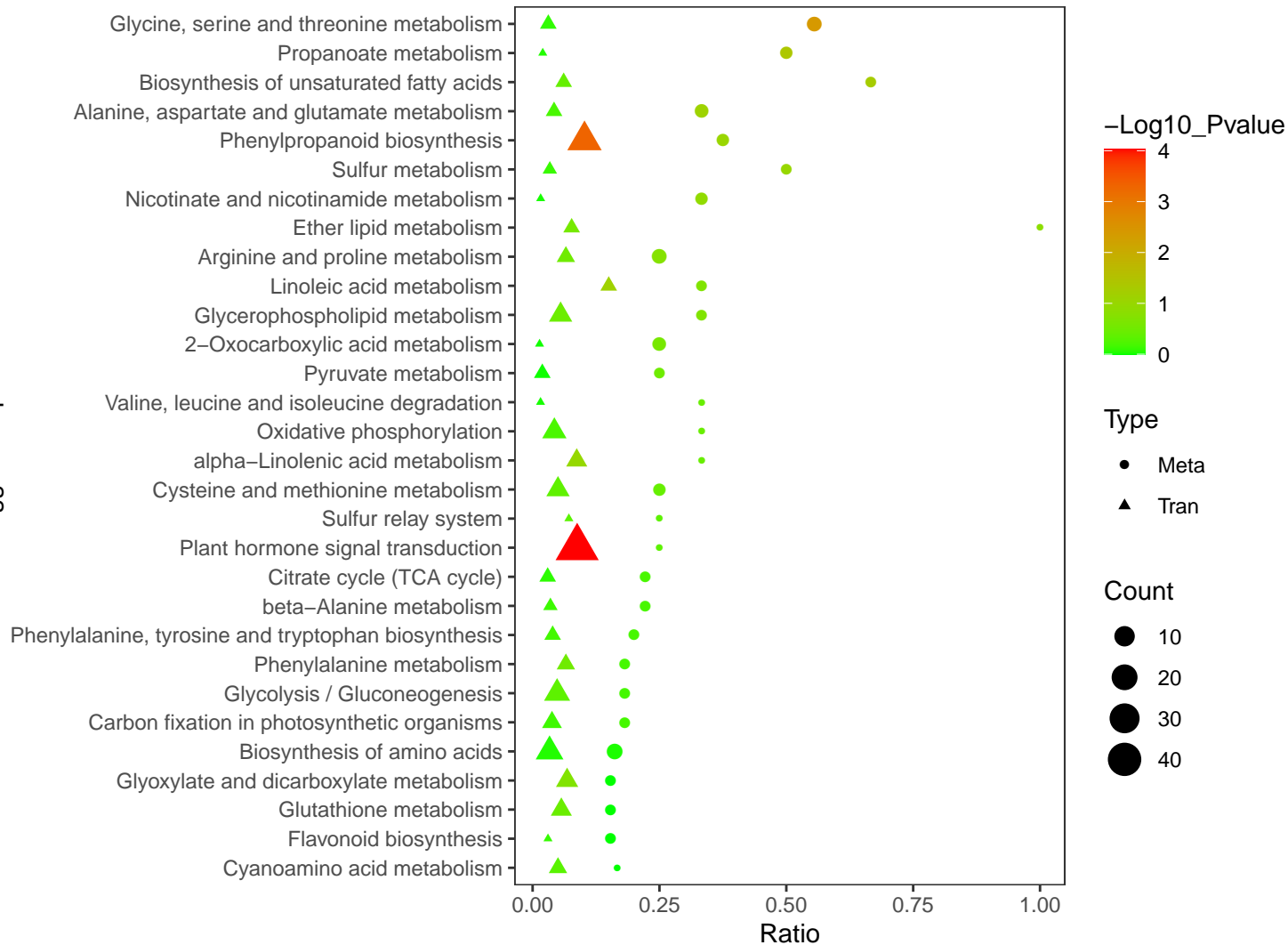

Supplement: Supplementary file 7 — Supplementary Figure S6. [file 41598_2023_40918_MOESM7_ESM.pdf]

# C.vs.A

kegg description

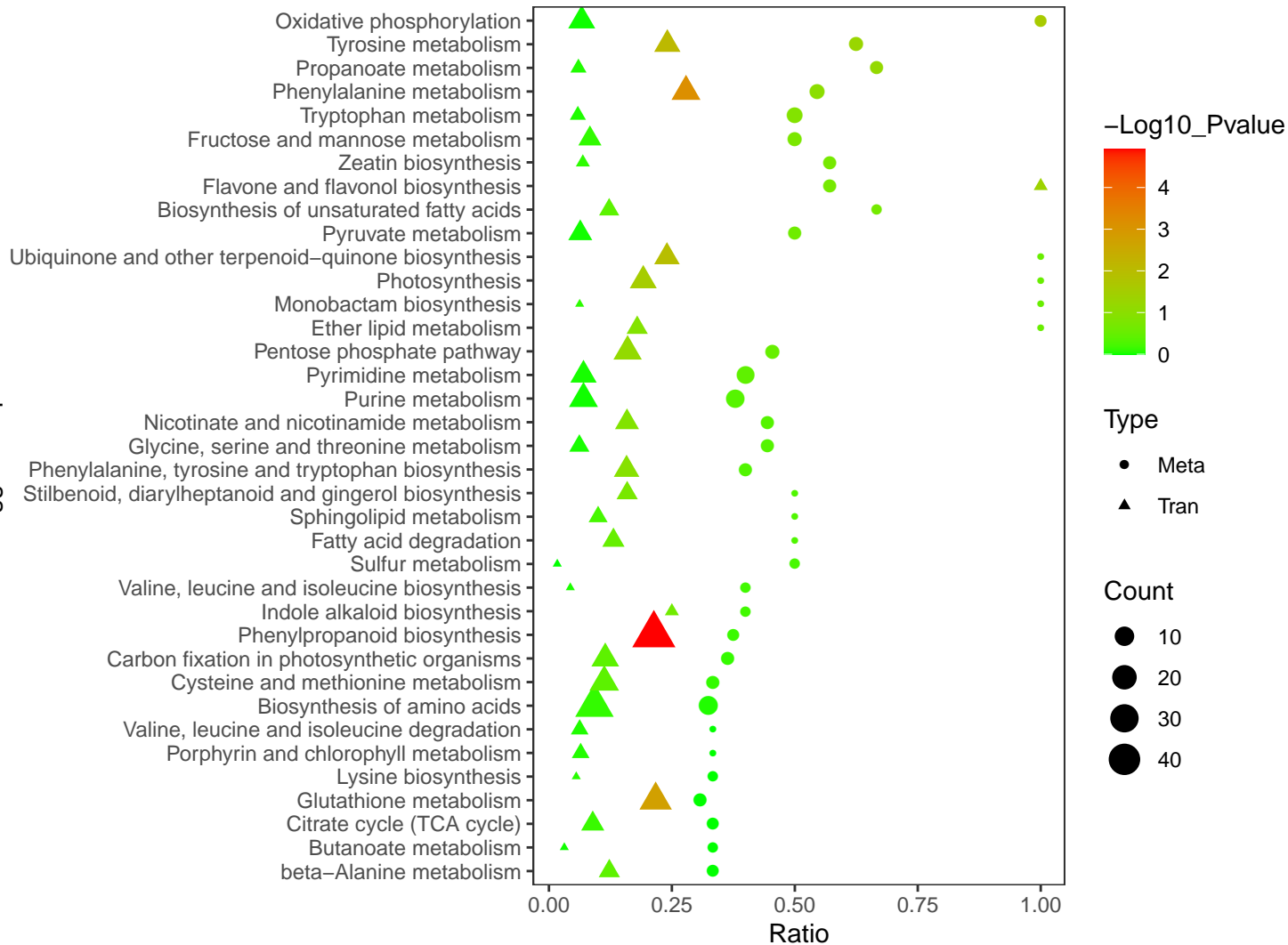

Supplement: Supplementary file 8 — Supplementary Figure S7. [file 41598_2023_40918_MOESM8_ESM.pdf]

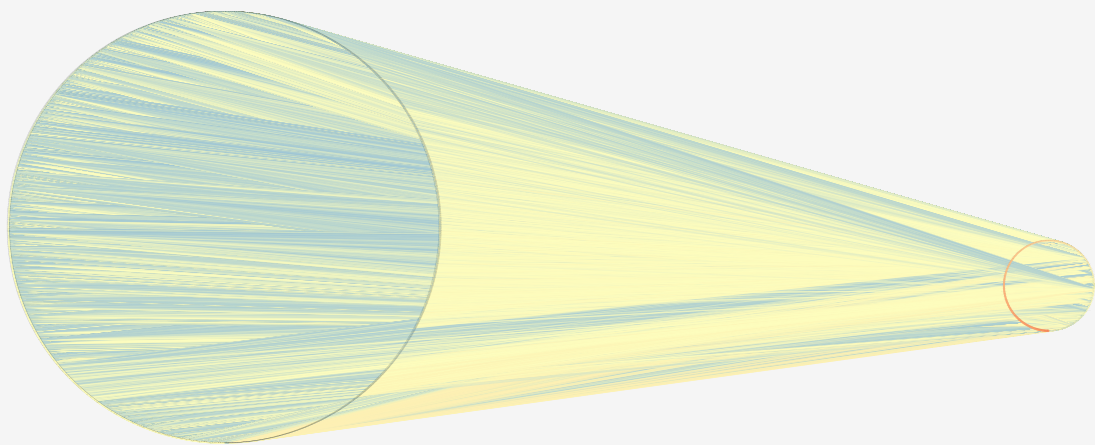

Supplement: Supplementary file 9 — Supplementary Figure S8. [file 41598_2023_40918_MOESM9_ESM.pdf]

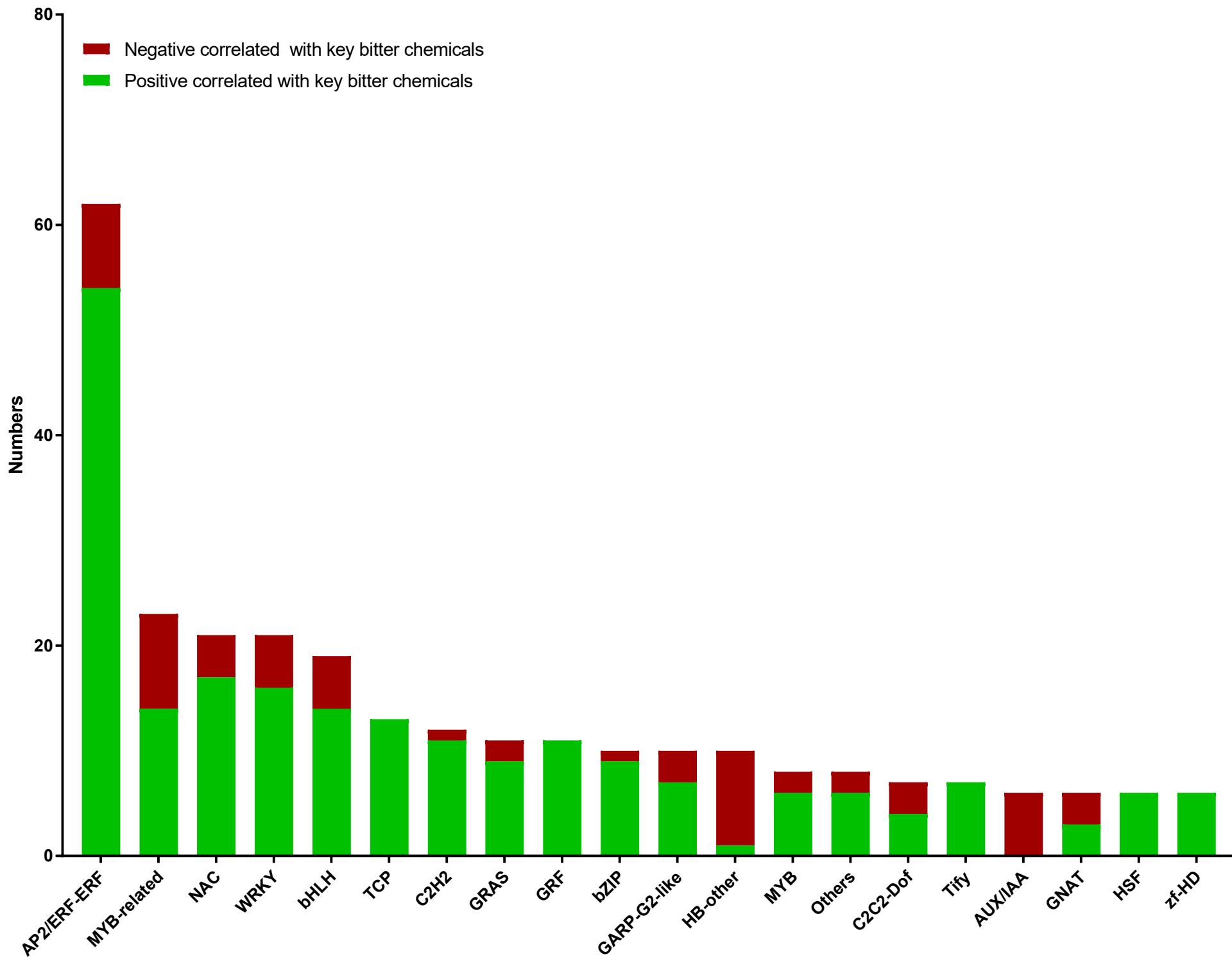

Supplement: Supplementary file 10 — Supplementary Figure S9. [file 41598_2023_40918_MOESM10_ESM.pdf]
